# Molecular and cytological features of the mouse B-cell lymphoma line iMyc^Eμ^-1

**DOI:** 10.1186/1476-4598-4-40

**Published:** 2005-11-09

**Authors:** Seong Su Han, Arthur L Shaffer, Liangping Peng, Seung Tae Chung, Jae Hwan Lim, Sungho Maeng, Joong Su Kim, Nicole McNeil, Thomas Ried, Louis M Staudt, Siegfried Janz

**Affiliations:** 1 Laboratory of Genetics, Center for Cancer Research (CCR), National Cancer Institute (NCI), NIH, Bethesda, MD, USA; 2 Metabolism Branch, CCR, NCI, NIH, Bethesda, MD, USA; 3 Laboratory of Metabolism, CCR, NCI, NIH, Bethesda, MD, USA; 4 Laboratory of Cellular Carcinogenesis and Tumor Promotion, CCR, NCI, NIH, Bethesda, MD, USA; 5 Genetics Branch, CCR, NCI, NIH, Bethesda, MD, USA; 6 Korea Research Institutes of Bioscience and Biotechnology, Daejeon (J. S. K.) and Department of Biological Sciences, Andong National University, Andong, South Korea (J. H. L.)

## Abstract

**Background:**

*Myc*-induced lymphoblastic B-cell lymphoma (LBL) in iMyc^Eμ ^mice may provide a model system for the study of the mechanism by which human *MYC *facilitates the initiation and progression of B cell and plasma cell neoplasms in human beings. We have recently shown that gene-targeted iMyc^Eμ ^mice that carry a His_6_-tagged mouse *Myc *cDNA, *Myc*^His^, just 5' of the immunoglobulin heavy-chain enhancer, Eμ, are prone to B cell and plasma cell tumors. The predominant tumor (~50%) that arose in the iMyc^Eμ ^mice on the mixed genetic background of segregating C57BL/6 and 129/SvJ alleles was LBL. The purpose of this study was to establish and characterize a cell line, designated iMyc^Eμ^-1, for the in-depth evaluation of LBL *in vitro*.

**Methods:**

The morphological features and the surface marker expression profile of the iMyc^Eμ^-1 cells were evaluated using cytological methods and FACS, respectively. The cytogenetic make-up of the iMyc^Eμ^-1 cells was assessed by spectral karyotyping (SKY). The expression of the inserted *Myc*^His ^gene was determined using RT-PCR and qPCR. Clonotypic immunoglobulin gene arrangements were detected by Southern blotting. The global gene expression program of the iMyc^Eμ^-1 cells and the expression of 768 "pathway" genes were determined with the help of the Mouse Lymphochip^© ^and Superarray^© ^cDNA micro- and macroarrays, respectively. Array results were verified, in part, by RT-PCR and qPCR.

**Results:**

Consistent with their derivation from LBL, the iMyc^Eμ^-1 cells were found to be neoplastic IgM^high^IgD^low ^lymphoblasts that expressed typical B-cell surface markers including CD40, CD54 (ICAM-1), CD80 (B7-1) and CD86 (B7-2). The iMyc^Eμ^-1 cells harbored a reciprocal T(9;11) and three non-reciprocal chromosomal translocations, over-expressed *Myc*^His ^at the expense of normal *Myc*, and exhibited gene expression changes on Mouse Lymphochip^© ^microarrays that were consistent with *Myc*^His^-driven B-cell neoplasia. Upon comparison to normal B cells using eight different Superarray^© ^cDNA macroarrays, the iMyc^Eμ^-1 cells showed the highest number of changes on the NFκB array.

**Conclusion:**

The iMyc^Eμ^-1 cells may provide a uniquely useful model system to study the growth and survival requirements of *Myc*-driven mouse LBL *in vitro*.

## Background

Gene-targeted iMyc^Eμ ^mice contain a single-copy mouse *Myc*^His ^(c-*myc*) cDNA that has been inserted in opposite transcriptional orientation in the mouse immunoglobulin heavy-chain gene cluster, *Igh*. The specific insertion site of the *Myc*^His ^transgene is in the intervening region of the *Igh *joining gene locus, J_H_, and the intronic heavy-chain enhancer, Eμ. The inserted transgene encodes a C-terminal His_6 _tag that is useful to distinguish message and protein encoded by *Myc*^His ^and normal *Myc *[1]. The iMyc^Eμ ^mice provide a model system for the study of the molecular and oncogenic consequences of the human *MYC- *and mouse *Myc*-deregulating chromosomal t(8;14)(q24;q32) and T(12;15) translocations that are widely accepted as the crucial initiating oncogenic events in the great majority of human Burkitt lymphomas (BL) and mouse plasmacytomas, respectively [2]. Specifically, the iMyc^Eμ ^mice mimic the type of t(8;14)(q24;q32) and T(12;15) translocation that is found in the endemic form of BL [3] and a subset (~20%) of IL-6 transgenic mouse plasmacytomas [4], respectively. We have recently shown that heterozygous transgenic iMyc^Eμ ^mice on the mixed genetic background of segregating C57BL/6 and 129/SvJ alleles are genetically prone to mature B cell and plasma cell neoplasms, ~50% of which are IgM^+ ^lymphoblastic B-cell lymphomas (LBL) [1]. We now report on a newly established LBL-derived cell line, iMyc^Eμ^-1, which was developed to study the growth and survival requirements of LBL *in vitro*.

## Results and Discussion

### Features of iMyc^Eμ^-1 cells

**Figure 1:**
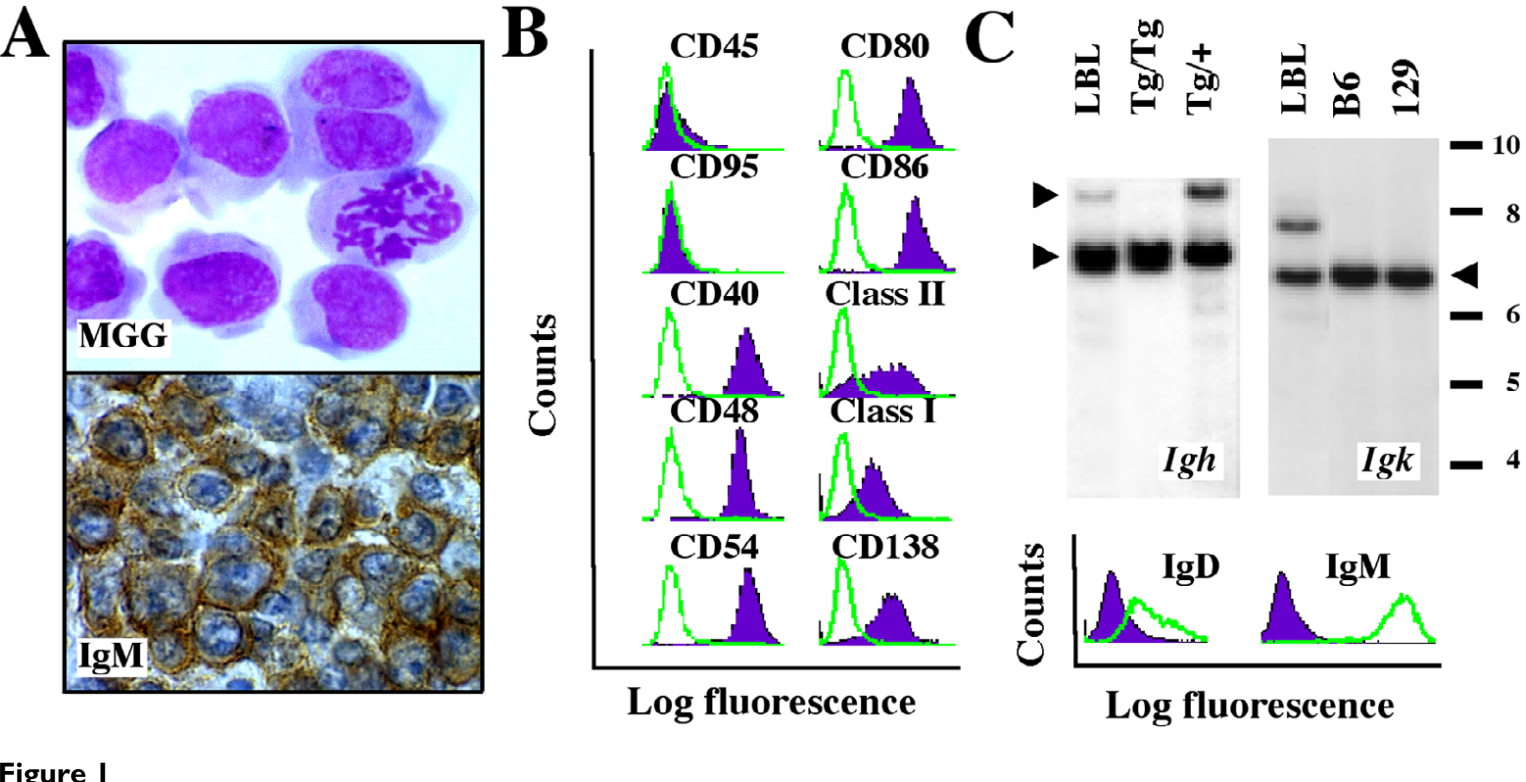
**Features of iMyc^Eμ^-1 cells**. *A*, cytofuge specimen of cultured cells stained according to May-Grünwald-Giemsa (top). Tissue section of the LBL from which the cell line was derived after immunostaining for μ H-chain (bottom). *B*, B-cell surface marker expression evaluated by FACS in cells treated with specific antibodies (purple histograms) or isotype controls (green lines). *C*, H/L rearrangements and surface Ig expression. Southern blots of *Igh *(top left) and *Igk *(top right) rearrangements of the LBL from which the cell line was derived. Included as control is liver DNA from homozygous (Tg/Tg) or heterozygous (Tg/+) transgenic iMyc^Eμ ^mice (left panel) or inbred C57BL/6 and 129SvJ mice (right panel). Recombination at the *Igh *locus was detected by the reduction of the normal, H chain-encoding upper fragment (upper arrowhead) in the face of comparable amounts of the mutated, *Myc*^His^-harboring lower fragment (lower arrowhead). Thus, the 6.2 kb long upper fragment was diminished in the LBL compared to the Tg/+ sample (and absent, as expected, in the Tg/Tg sample), whereas the *Myc*^His^-harboring lower fragment was comparable. The *Myc*^His^-bearing *Igh *locus cannot encode H chain because of the gene insertion. Recombination at the *Igκ *locus resulted in an enlarged fragment (~7.8 kb) compared to the germ line fragment that is indicated by the arrowhead pointing left. Detection of surface IgM^hi^IgD^low ^using FACS analysis (bottom).

**Figure 2:**
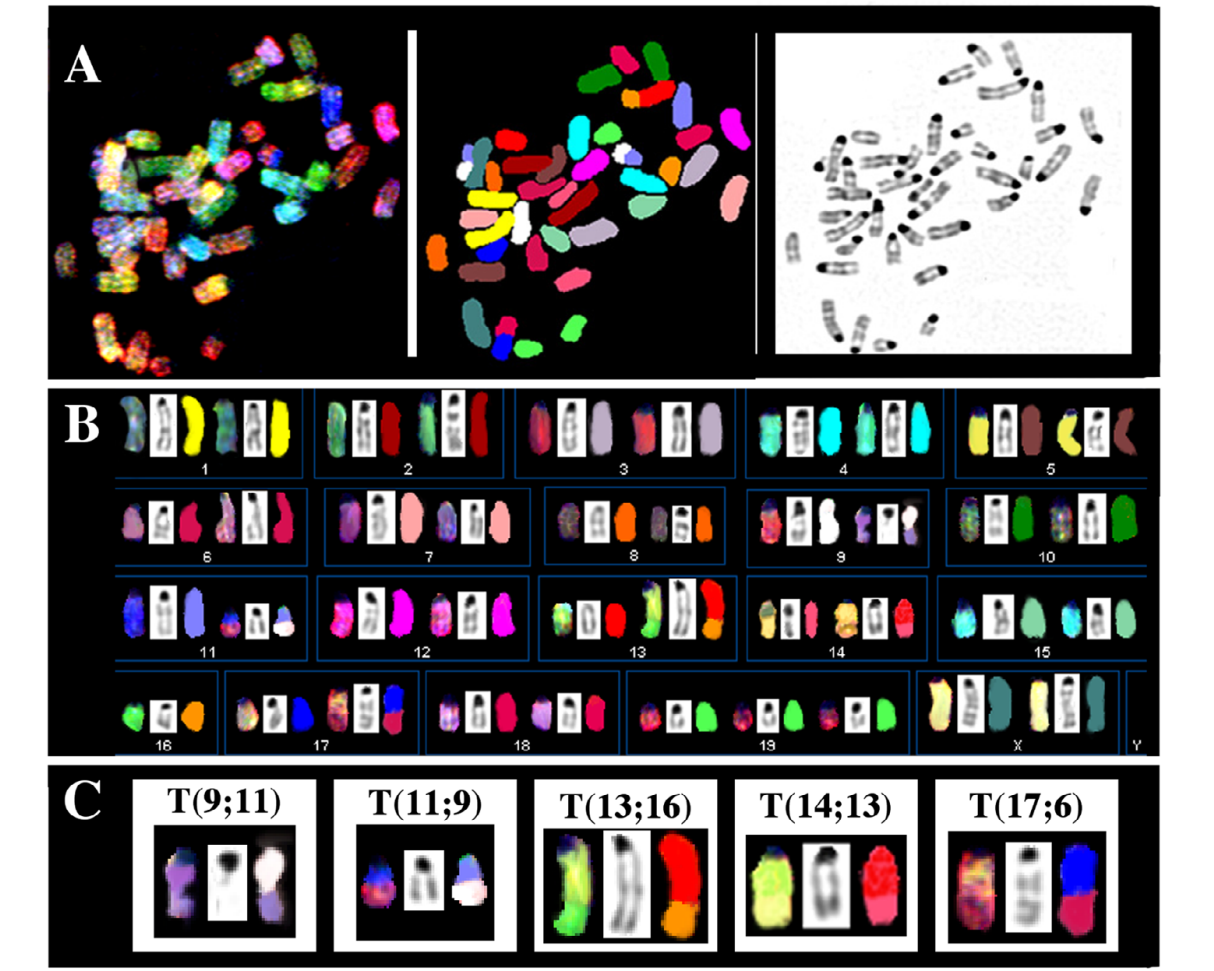
**Spectral karyotype of iMyc^Eμ^-1 cells**. *A*, representative metaphase chromosome spread in SKY display (left) and classification colors (center) and as an inverted DAPI image (right). *B*, complete, near-diploid tumor karyotype depicting each chromosome in SKY display (left) and classification (right) colors and after staining with DAPI (center): 38–40, XX, Del(4)(C4)[2], Del(6)(D2)[6], Der(9)T(9A4;11E2)[4], Der(11)T(11A4;9E4)[4], Del(12)[3], Der(13)T(13D11;16B5)[5], Der(14)T(14;13)[3], Der(17) T(17D;6D3)[5], +19[6]. The chromosomes are arranged in numerical order from left to right and top to bottom. *C*, chromosomal translocations that took the form of a reciprocal T(9;11) exchange (left) and three different non-reciprocal exchanges (right).

The iMyc^Eμ^-1 cell line, which demonstrated the typical cytological features of mouse LBL (Fig. 1A top), was derived from a primary IgM^+^LBL (Fig. 1A bottom) that exhibited moderate plasmacytic differentiation potential *in situ *(not shown). FACS analysis using a panel of antibodies to B cell surface markers (Fig. 1B) showed that iMyc^Eμ^-1 cells were positive for CD40, CD48, CD54, CD80/86 (B7-1/2) and CD138 (syndecan 1). Expression of class I and II MHC antigens and CD45 (B220) was detectable at low or very low levels, respectively, but CD95 (Fas) was absent. Treatment of iMyc^Eμ^-1 cells with antibody to CD40 led to the induction of Fas and upregulation of CD45 and CD54, activation markers CD80/86, and CD138, indicating that CD40 signaling was functional (Additional File 1). Southern blotting of genomic DNA from the LBL from which the cell line was derived demonstrated V(D)J rearrangement at the Ig heavy-chain and κ light chain loci (Fig. 1C top). The expression of surface IgM^high^IgD^low ^by the derivative cell line was consistent with this and indicated that the rearrangement was productive (Fig. 1C bottom). SKY analysis of metaphase chromosomes from iMyc^Eμ^-1 cells (Fig. 2) uncovered four chromosomal translocations that took the form of a reciprocal T(9;11) exchange and three non-reciprocal exchanges: T(13;16), T(14;13) and T(17;6). Although it is unclear whether these translocations occurred during tumor development or establishment of the cell line [5], repeat karyotyping showed that the present iMyc^Eμ^-1 line is cytogenetically stable.

### Gene expression profile of iMyc^Eμ^-1 cells on cDNA microarray

The Mouse Lymphochip, a microarray of hematopoietic mouse cDNA clones, provides a powerful tool to evaluate the similarity of primary mouse B cells, B-cell tumors, and tumor-derived cell lines at the level of global gene expression [6]. To compare the gene expression profile of LBL and iMyc^Eμ^-1 cells, RNA was obtained from primary B cells, fresh-frozen tumors and iMyc^Eμ^-1 cells. The RNA was labeled with Cy5-dUTP, and hybridized to the cDNA microarray spotted on a glass slide. An RNA control pool labeled with Cy3-dUTP was co-hybridized to the same array and used as a common denominator by which all samples were compared to one another. Further information on microarray make-up, analysis and data interpretation is available at: .

**Figure 3:**
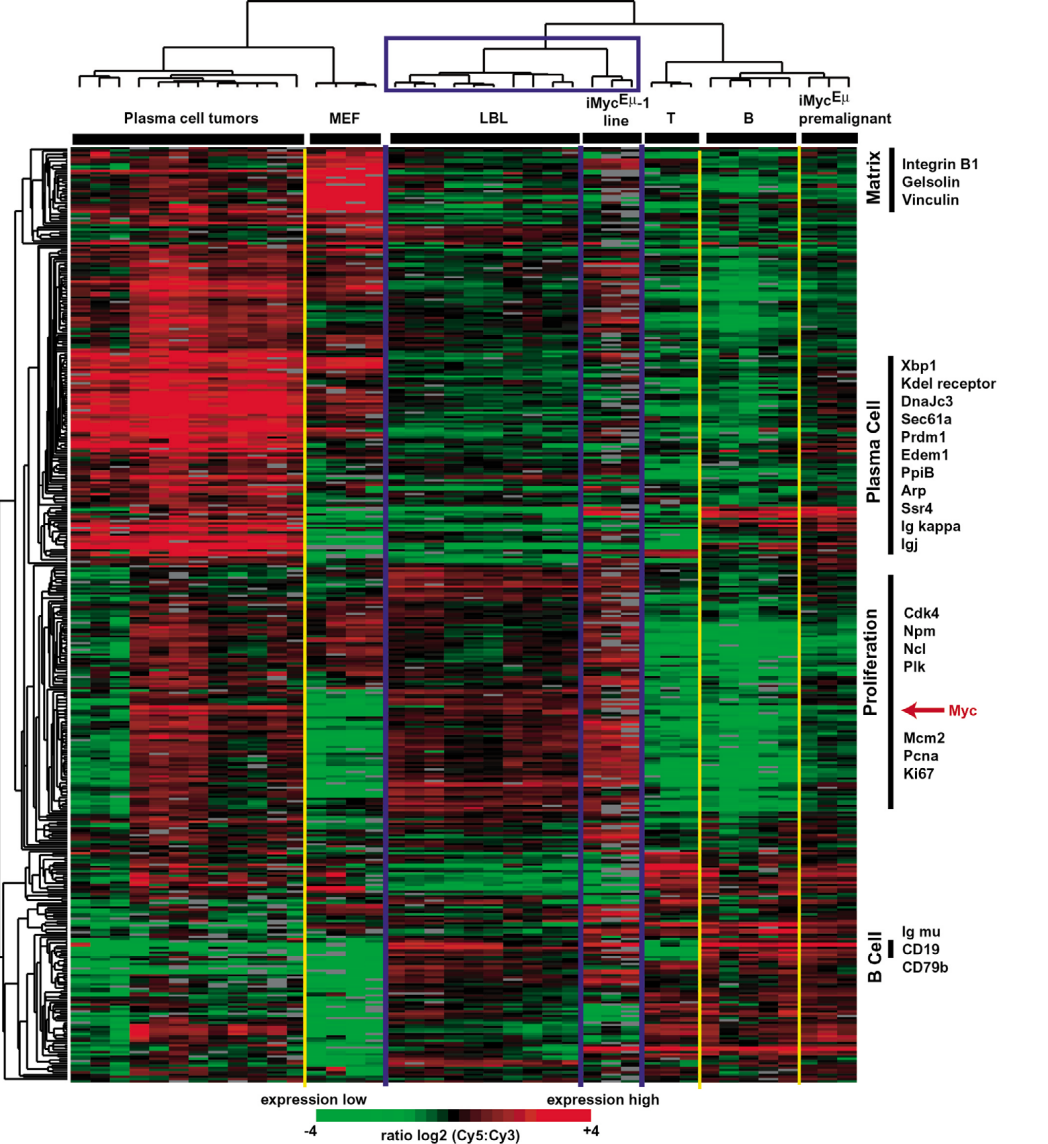
Similar gene expression profile of iMyc^Eμ^-1 cells and LBL using comparative cDNA microarray measurements. Relative gene expression levels are depicted according to the color scale shown below the cluster.

Three independent RNA samples of iMyc^Eμ^-1 cells and ten primary LBL were analyzed together with a collection of normal, resting mouse B cells and T cells, mouse embryonic fibroblasts (MEF), and peritoneal plasmacytomas that arose in pristane-treated BALB/c mice. A total of 414 well-characterized array elements that clustered across these samples based on gene expression patterns (Fig. 3) demonstrated a clear distinction of lymphoid and non-lymphoid cell types (B and T cells versus MEF), lymphocyte lineages (B versus T cells), and transformation- and development-associated differences within the B-cell lineage (normal B cells versus LBL and PCT). The gene expression profiles of LBL and iMyc^Eμ^-1 cells, which clustered in one tight group (Fig. 3 top, blue rectangle), exhibited a remarkable homogeneity. Compared to the plasmacytomas, LBL and iMyc^Eμ^-1 cells maintained many hallmark genes in the B cell signature (e.g., those encoding CD19, CD79 and μ heavy-chain) but under-expressed numerous genes in the plasma cell signature (e.g., *Sec61*, *Ssr4 *and *DNAjc3*) and matrix signature (e.g., those encoding vinculin, gelsolin and integrin B1) [7,8]. A more detailed analysis of the plasma cell signature revealed that in contrast to *Xbp1 *and its target genes, the iMyc^Eμ^-1 cells expressed *Prdm1 *(*Blimp1*). This suggested that the cells underwent neoplastic transformation at the early stage of plasmacytic differentiation (Additional File 2).

### Myc expression in LBL and iMyc^Eμ^-1 cells

**Figure 4:**
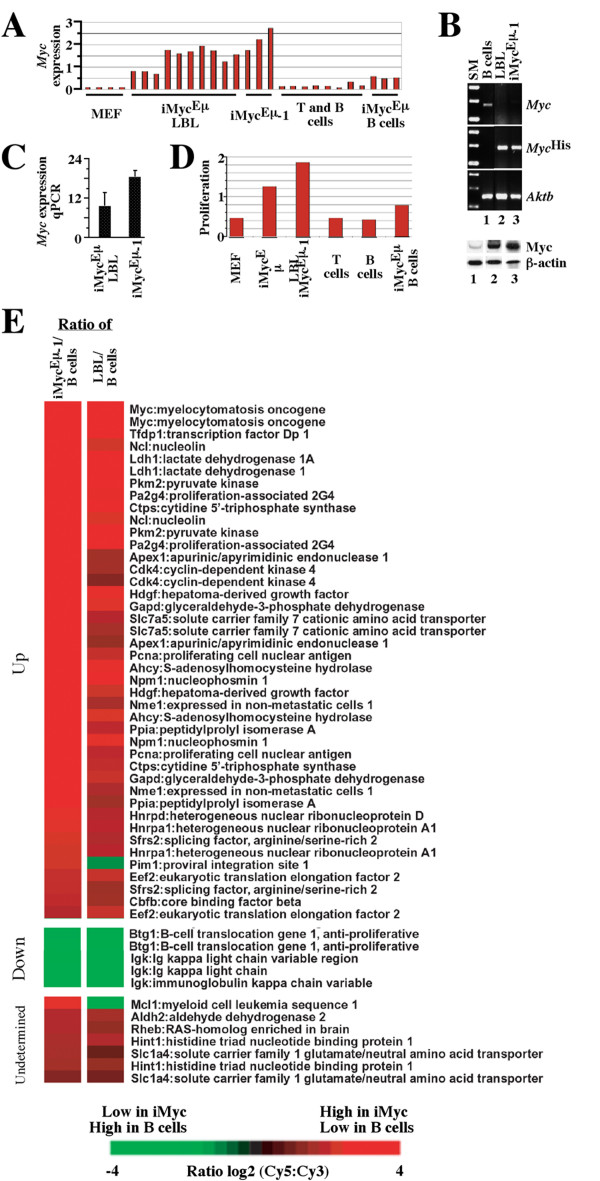
***Myc *expression and *Myc*-driven gene expression changes in iMyc^Eμ^-1 cells**. *A*, expression of *Myc *in iMyc^Eμ^-1 cells, LBL, and normal controls. The average gene expression was calculated and plotted according to the microarray measurements shown in Figure 3. *B*, RT-PCR of *Myc*, *Myc*^His ^and *Aktb *mRNA levels (top) and Western blotting of Myc protein (bottom) in normal B cells (lane 1), LBL from an iMyc^Eμ ^mouse (lane 2), and the iMyc^Eμ^-1 cell line (lane 3; SM, size marker). *C*, real-time qPCR analysis of *Myc *mRNA levels in iMyc^Eμ^-1 and LBL cells. Mean values and standard deviations based on three independent iMyc^Eμ^-1 and five LBL samples are shown. *D*, expression of proliferation signature genes in iMyc^Eμ ^samples and normal controls. The expression of genes that fall in the proliferation signature defined in Figure 3 was averaged for each cell population and plotted. *E*, differentially expressed Myc targets in iMyc^Eμ^-1 cells (left column) and LBL (right column) compared to normal resting B cells. Relative gene expression levels are depicted according to the color scale at the bottom. Gene designations and names are listed to the right.

The expression levels of *Myc*, as measured by the arrays, was clearly elevated in LBL and iMyc^Eμ^-1, intermediate in "premalignant" B cells from tumor-free iMyc^Eμ ^mice, and absent, as expected, in resting lymphocytes from normal mice (Fig. 4A). Because the inserted *Myc *cDNA in iMyc^Eμ ^mice also encodes a C-terminal His_6 _tag, it is possible to distinguish message and protein encoded by *Myc*^His ^and normal *Myc*. Allele-specific RT-PCR analysis of *Myc*^His ^and *Myc *mRNA demonstrated that, in common with LBL, iMyc^Eμ^-1 cells expressed predominantly the transgene (Fig. 4B top, lanes 2–3). This pattern of suppression of the normal *Myc *gene [9] is also a feature of human B-cell lymphomas containing constitutively deregulated *MYC *[10]. Western blotting with an anti-Myc antibody detecting both Myc^His ^and normal Myc proteins (Fig. 4B bottom) showed that LBL and iMyc^Eμ^-1 cells over-expressed Myc at comparable levels (lanes 2–3) relative to B splenocytes from non-transgenic littermates (lane 1). To compare the levels of *Myc *in LBL and iMyc^Eμ^-1 cells more precisely, we performed qPCR using *Aktb *mRNA levels as internal standard. The iMyc^Eμ^-1 cells expressed nearly twice as much *Myc *as the LBL (Fig. 4C). The levels of *Myc *also correlated with the expression of genes from the proliferation cluster when the gene expression from the proliferation signature, as defined in Figure 3, was averaged for each cell type. Proliferation gene expression was low in unstimulated cells (MEF and B/T cell samples), intermediate in pre-malignant B cells from iMyc^Eμ ^mice, and upregulated in LBL and iMyc^Eμ^-1 (Fig. 4D).

### Myc target genes in LBL and iMyc^Eμ^-1 cells

To further examine the contribution of the *Myc*^His ^transgene to the gene expression profile of LBL and iMyc^Eμ^-1, we performed a statistical analysis (Student's T test) of genes differentially expressed between normal B cells versus LBL and iMyc^Eμ^-1 cells. A total of 122 array elements from Figure 3 were significantly differential in their expression when B cells were compared to iMyc^Eμ^-1 cells and LBL (*p *< 0.015, 1.5-fold minimal difference in average expression). The vast majority (97%) of these elements, many of them previously identified as proliferation-associated Myc targets , behaved similarly in both the cell line and LBL (Fig. 4E). Twenty-four known Myc targets were up-regulated in the LBL and iMyc^Eμ^-1 cells (*Ahcy, Apex1, Cbfb, Cdk4, Ctps, Eef2, Gapd, Hdgf, Hnrpa1, Hnrpd, Idh1, Myc, Ncl, Nme1, Npm1, Pa2g4, Pcna, Pim1, Pkm2, Ppia, Sfrs2Slc7a5*, *Tfdp1*), two were down-regulated (*Btg1, Igk*), and five were undetermined as to the effect of Myc on their expression (*Aldh2, Hint1, Mcl1, Rheb, Slc1a4*). These findings were in accordance with the nature of LBL and iMyc^Eμ^-1 as *Myc*-driven B-cell tumors and firmly established the similarity of LBL and iMyc^Eμ^-1at the level of a single gene (Fig. 4A), a gene expression signature (Fig. 4D), and globally (Fig. 3).

### Validation of gene expression changes in iMyc^Eμ^-1 cells

To further compare the gene expression profiles of LBL and iMyc^Eμ^-1, and validate the cDNA microarray results with an independent method, we used cDNA macroarrays on nylon membranes to assess the expression of selected "pathway" genes in LBL, iMyc^Eμ^-1 and normal B cells. Included in the analysis were RNA samples of iMyc^Eμ^-1 and LBL previously analyzed on the microarray. Freshly prepared RNA from normal, MACS purified, B220^+ ^splenocytes were used as control. RNA samples were labeled with ^32^P-dUTP and individually hybridized to the macroarrays. Individual expression profiles were determined and compared with each other. Reproducible two-fold or higher changes in hybridization signal intensity were used as threshold for gene expression changes. Eight different macroarrays, each containing 96 genes involved in cell cycle regulation, apoptosis, cancer, signal transduction, stress and toxicity responses, and the NFκB and MAPK pathways, were used. The primary data set is depicted in Additional File 3.

**Figure 5:**
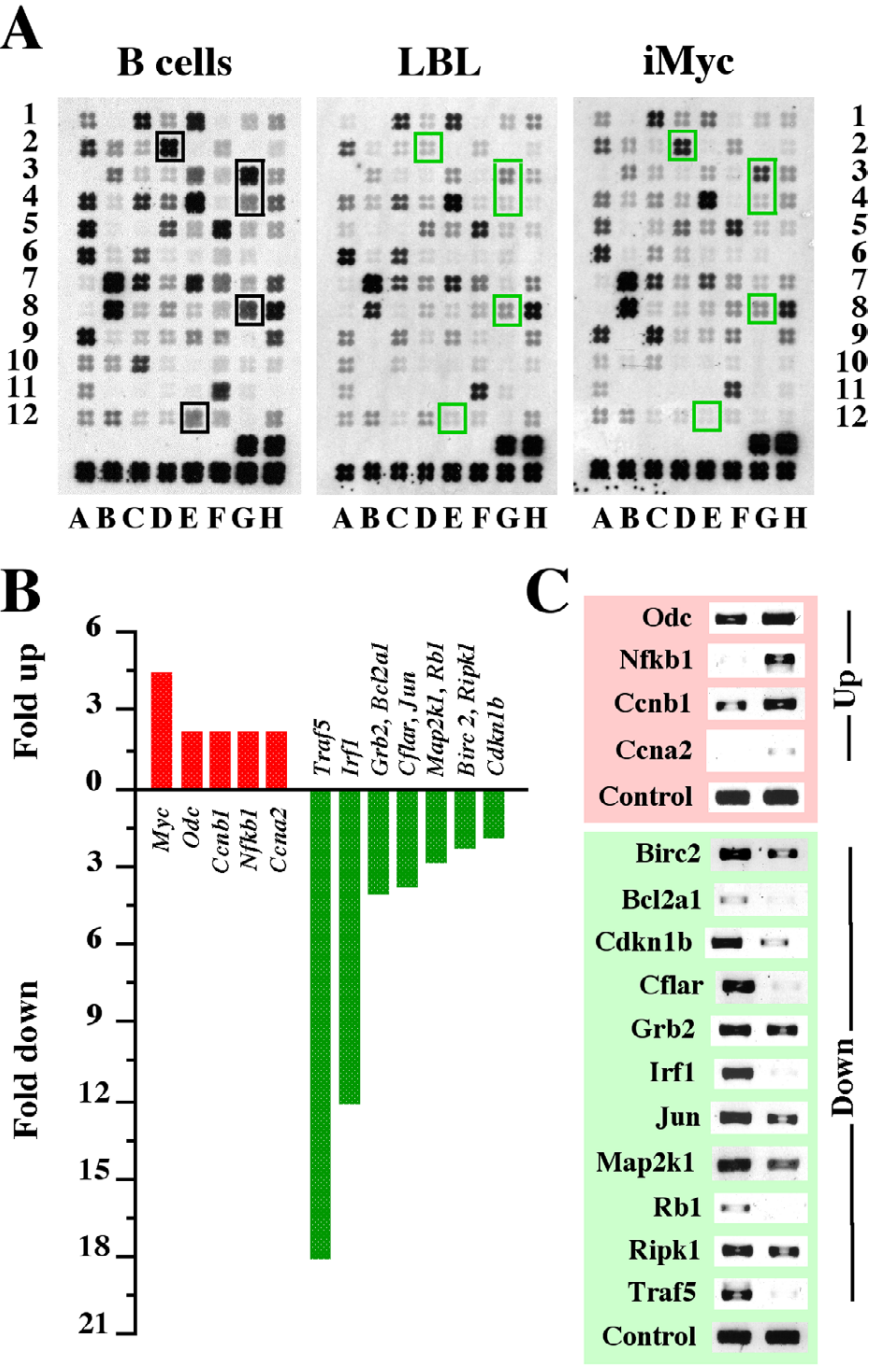
**Concordant gene expression changes in iMyc^Eμ^-1 cells and LBL compared to normal B cells**. *A*, gene expression changes were assessed by comparative filter cDNA macroarray measurements using a representative apoptosis array of normal B cells (left), LBL (center) and iMyc^Eμ^-1 cells (right) as the example. Compared to B cells, LBL and iMyc^Eμ^-1 cells under-expressed *Bcl2a1d *(array position D2), *Birc2 *(G3), *Cflar *(G4), *Ripk1 *(G8) and *Traf5 *(E12; indicated by green squares). Additional File 3 shows four additional LBL arrays that exhibit the same changes. *B*, expression changes of 5 up-regulated and 11 down-regulated genes in iMyc^Eμ^-1 cells compared to normal B cells (see Additional File 6 for names, functions and groupings of these genes). Similar changes were seen when LBL and B cells were compared using cDNA macroarrays (not shown) or when iMyc^Eμ^-1 cells and/or LBL were compared to B cells using cDNA microarrays (Fig. 3; results not shown). *C*, verification of gene array results using RT-PCR. Shown are ethidium bromide-stained PCR fragments of the differentially regulated genes plotted in panel B except *Myc*, which was verified in the experiments presented in Figure 4A-C. The iMyc^Eμ^-1 and B-cell samples are shown in the right and left lane, respectively. Up and down regulated genes are depicted on the pink and green background, respectively.

The changes on the macroarrays were remarkably consistent in LBL and iMyc^Eμ^-1 cells compared to B cells. This is illustrated in Figure 5A, using the apoptosis array as the example. Among a total of 768 genes present on eight different macroarrays, 121 (16%) genes were concordantly up- or down-regulated in LBL and iMyc^Eμ^-1 cells relative to B cells. The NFκB array showed the highest number of changes (n = 22) among the eight different macroarrays, followed by the MAPK (n = 19), cell cycle (n = 18) and apoptosis arrays (n = 17). The stress and toxicity array contained the lowest number of changes (n = 9). Altogether, down-regulated genes (83/121, 69%) outnumbered up-regulated genes (38/121, 31%) by a factor of 2.2. The presence of some genes on two or more arrays afforded an opportunity for additional quality control. Genes of that sort exhibited the same trend on different arrays, either up or down relative to normal B cells, thus adding confidence in the results. This is illustrated in Additional File 4 using one representative array each of iMyc^Eμ^-1 and normal B cells.

Among the differentially regulated genes that exhibited concordant changes in LBL and iMyc^Eμ^-1 cells relative to normal B cells on both the gene micro- and macroarrays were 16 genes that were selected for further confirmation using semi-quantitative RT-PCR (Additional File 5). These genes were of particular interest to us because of possible follow-up studies on signaling pathways in iMyc^Eμ^-1 cells. Eleven of the 16 genes were down regulated (*Bcl2a1, Birc2, Cflar, Cdkn1b, Grb2, Irf1, Jun, Map2k1, Rb1, Ripk1, Traf5*) and five genes were up regulated (*Ccna2, Ccnb, Myc, Nfkb1, Odc*). Figure 5B presents average quantitative changes on the macroarrays when iMyc^Eμ^-1 cells were compared with normal B cells. These changes were readily confirmed by RT-PCR in all cases (Fig. 5C) except *Myc*, which was not included because it was confirmed in previous work (Fig. 4).

## Conclusion

This study reports the molecular, cytogenetic and morphological features of a stable cell line, designated iMyc^Eμ^-1. The iMyc^Eμ^-1 cells are surface IgM^high^IgD^low ^and cytogenetically stable using SKY. The cells express high levels of the inserted *Myc*^His ^transgene and exhibit a global gene expression profile consistent with that of *Myc*^His^-driven B-cell neoplasia. The iMyc^Eμ^-1 cells may be useful for in-depth studies on the growth and survival requirements of *Myc*^His^-driven mouse B-cell tumors *in vitro*. Specifically, the cells may facilitate the elucidation of the signal transduction pathways that appear to maintain high Myc protein levels in mouse LBL [1]. These studies may results in new approaches to treat and prevent *MYC*-induced B cell and plasma cell neoplasms in human beings.

## Methods

### Mouse lymphomas and derivation of iMyc^Eμ^-1 cells

Transgenic iMyc^Eμ ^mice develop a high incidence of B cell and plasma cell tumors with LBL being the predominant phenotype (8). Tumor samples obtained at autopsy were fixed in formalin for later histopathology or frozen for later preparation of protein, DNA and RNA. Histological criteria used for diagnosing mouse LBL are detailed elsewhere [11]. Highly enriched splenic B cells were prepared from C57BL/6 mice using CD45R (B220) microbeads and MACS separation columns (Miltenyi Biotec, Auburn, CA). All mice were maintained under Animal Study Protocol LG-028. The iMyc^Eμ^-1 cell line was derived from a LBL and maintained at 37°C and 5% carbon dioxide in RPMI 1640 medium supplemented with 10% fetal calf serum, 200 mM L-glutamine, 50 μM 2-mercaptoethanol and penicillin/streptomycin (Gibco-BRL, Rockville, MD).

### Characterization of iMyc^Eμ^-1 cells

For cytological analysis, cytofuge specimens were stained according to May-Grünwald-Giemsa and inspected by microscopy. For detection of chromosomal aberrations, cells were analyzed by spectral karyotyping (SKY) as previously described [12]. For flow cytometry, single-cell suspensions were stained and analyzed on a FACSort^® ^using the CELLQuest™ software (BD Pharmingen, San Diego, CA). Rat anti-mouse CD16/CD32 was used to block FcγII and FcγIII receptors. Antibodies to mouse CD45 (catalog number 553076), CD80 (553766), Fas (CD90, 554255), CD86 (553689), CD40 (553787), I-A^b ^(MHC class II, 553551), H-2K^b ^(MHC class I, 553569), CD48 (557483), CD54 (553250), CD138 (553712), IgD (553438) and IgM (53519) were purchased from BD Biosciences. For the evaluation of surface marker changes upon ligation of CD40, cells were incubated with rat anti-mouse CD40 (553787) using 3.5 μg antiboy per 5 × 10^5 ^cells. For Southern blot hybridization of clonotypic V(D)J rearrangements, genomic DNA (20 μg) was digested with BamHI and EcoRI, fractionated on a 0.7 % agarose gel, transferred to a nylon membrane, and crosslinked under UV light. Following pre-hybridization (Hybrisol I, Intergen) at 42°C, the membrane was hybridized to a 1.5-kb HindIII/EcoRI fragment of *Igh *spanning *J*_*H*_*2 *and *Eμ *or to a 1.1-kb Cκ probe, which was generated by PCR using a primer pair obtained from Dr. Michael Kuehl (NCI): 5'-GAT GCT GCA CCA ACT GTA TCC A-3' and 5'-GGG GTG ATC AGC TCT CAG CTT-3'. Probes were labeled with [^32^P]-CTP using a random priming kit.

### Allele-specific RT-PCR of Myc and Myc^His ^mRNA

For semi-quantitative determination of *Myc *and *Myc*^His ^mRNA, total RNA was isolated using TRIzol (Sigma, St. Louis, MO, USA). Double stranded cDNA was synthesized from 1 μg of total RNA, using the AMV Reverse Transcriptase kit (Roche, Indianapolis, IN). A common 5' primer for both *Myc*^His ^and *Myc *(5'-TCT CCA CTC ACC AGC ACA AC-3') was combined with a specific 3' primer for *Myc*^His ^(5'-CCT CGA GTT AGG TCA GTT TA-3') and *Myc *(5'-ATG GTG ATG GTG ATG ATG AC-3') to distinguish the two messages. Thermal cycling conditions were as follows: 95°C for 5 min followed by 20 cycles of amplification at 57°C, 72°C and 95°C, each for 1 min. PCR amplification of *Aktb *cDNA was performed as control using the following primer pair: 5'-GCA TTG TTA CCA ACT GGG AC-3' and 5'-AGG CAG CTC ATA GCT CTT CT-3'. PCR products were analyzed by electrophoresis in 1% agarose gel and visualized by staining with ethidium bromide.

### Real-time qPCR of Myc mRNA

For quantitative Taqman RT-PCR of Myc (*Myc *plus *Myc*^His^), total RNA was isolated from cells using TRIzol Reagent (Invitrogen). Serial dilutions of input RNA (100 ng - 1.56 ng) were analyzed in triplicates using the ABI PRISM 7900HT sequence detector system, primers, probes, and the Taqman One-Step RT-PCR Master Mix Reagents kit, all purchased from Applied Biosystems. The reaction mixture was held at 48°C for 30 min for reverse transcription of RNA into cDNA. This was followed by incubation at 95°C for 10 min to activate the Taq polymerase. PCR amplification of cDNA was performed for 40 cycles using the following cycling conditions: denaturing for 15 s at 95°C and annealing and extending for 1 min at 60°C. All samples were tested in triplicates, and average values were used for quantification. Analysis was performed using SDS v2.1 software (Applied Biosystems) according to the manufacturer's instruction. *Aktb *was used as internal reference gene. The comparative CT method (ΔΔCT) was used for quantification of gene expression.

### Gene microarray hybridization and analysis

cDNA made from total RNA (50 μg) from each tumor, primary cell sample, or iMyc^Eμ^-1 cells was labeled with cyanine 5-conjugated dUTP (Cy5). cDNA made from pooled mouse cell line RNA (50 μg) was labeled with cyanine 3-conjugated dUTP (Cy3) and used as reference. Microarray hybridizations were performed on Mouse Lymphochip microarrays [7]. After washing, the slides were scanned using an Axon GenePix 4.0 scanner (Axon Instruments Inc., Union City, CA). After normalization, those elements that failed to meet confidence criteria based on signal intensity and spot quality were excluded from analysis. In addition, data were discarded for any gene for which measurements were missing on >30% of the arrays or were not sequence-verified. The Cy5:Cy3 intensity ratios of the remaining spots were log_2 _transformed. To compare normal samples, hierarchical cluster analysis was performed using the Gene Cluster and Treeview programs [8].

### Gene macroarray hybridization and analysis

The relative mRNA expression of genes involved in regulation of apoptosis, cell cycle progression, NFkB signaling, and cellular stress and toxicity responses was analyzed with GEArray (SuperArray Inc., Bethesda, MD) according to the manufacturer's protocol. Cells were treated for 24 hrs with 0.4 mM and 1 mM CDDO-Im, respectively, followed by preparation of total RNA using TriReagent (Sigma). Five μg from each sample were reverse transcribed into ^32^P-labeled cDNA using MMLV reverse transcriptase (Promega, Madison, WI) and ^32^P-dCTP (NEN, Boston, MA). The resulting cDNA probes were hybridized to gene-specific cDNA fragments spotted in quadruplicates on the GEArray membranes. After stringent washing of the arrays, the signal of the hybridized spots was measured with a STORM PhosphorImager (Molecular Dynamics, Sunnyvale, CA) and normalized to the signal of the housekeeping gene *Gapd*. Array results on six CDDO-Im inducible genes were validated using semi-quantitative RT-PCR.

### Gene array validation using RT-PCR

For semi-quantitative determination of mRNA levels, total RNA was isolated and double stranded cDNA was synthesized as described above for *Myc*. Information on PCR primers and thermal cycling conditions is available in Additional File 6. PCR products were analyzed by electrophoresis in 1% agarose gel and visualized by staining with ethidium bromide.

## Competing interests

The author(s) declare that they have no competing interests.

## Authors' contributions

Seong-Su Han determined gene expression using Superarray^© ^cDNA macroarrays. Arthur L. Shaffer and Louis M. Staudt evaluated global gene expression profiles using Mouse Lymphochip^© ^microarrays. Liangping Peng performed FACS studies. Seung-Tae Chung harvested and transplanted tumors, cultured cells, and prepared histo- and cytological specimens. Sungho Maeng and Jae-Hwan Lim validated gene array results using RT-PCR and qPCR. Joong-Su Kim performed Southern analysis and Nicole McNeil and Thomas Ried performed SKY analysis. Siegfried Janz designed the study and wrote and approved the article.

## Supplementary Material

Additional File 1 FACS histograms. Click here for file

Additional File 2 Heat map (panel A) and bar graph (panel B). Click here for file

Additional File 3 Images of cDNA gene arrays. Click here for file

Additional File 4 Images of cDNA gene arrays (panel A) and gene table (panel B). Click here for file

Additional File 5 Gene list. Click here for file

Additional File 6 PCR primers and conditions. Click here for file
