# Supplementary figures and images for: Molecular and cytological features of the mouse B-cell lymphoma line iMycEμ-1
Source: Mol Cancer. 2005 Nov 9;4:40. doi: 10.1186/1476-4598-4-40 (PMC1298327; doi:10.1186/1476-4598-4-40)

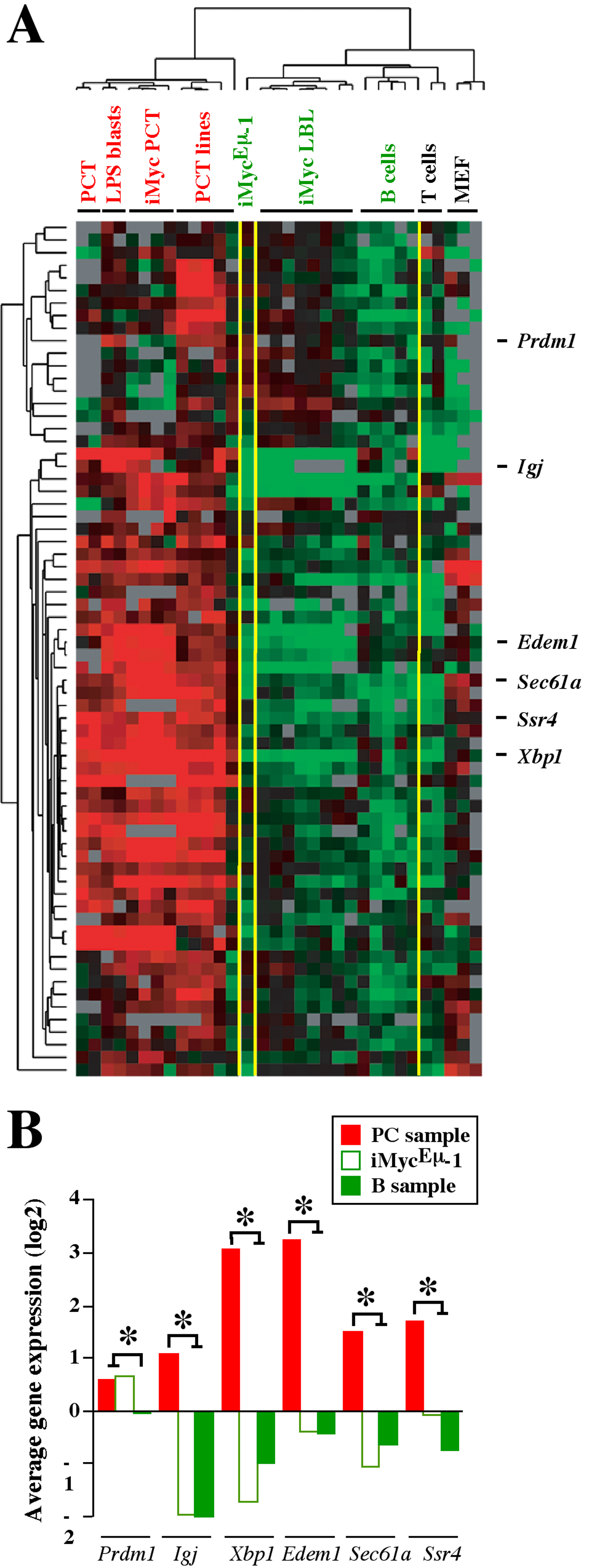

Supplement: Additional File 2 — Heat map (panel A) and bar graph (panel B). [file 1476-4598-4-40-S2.png]

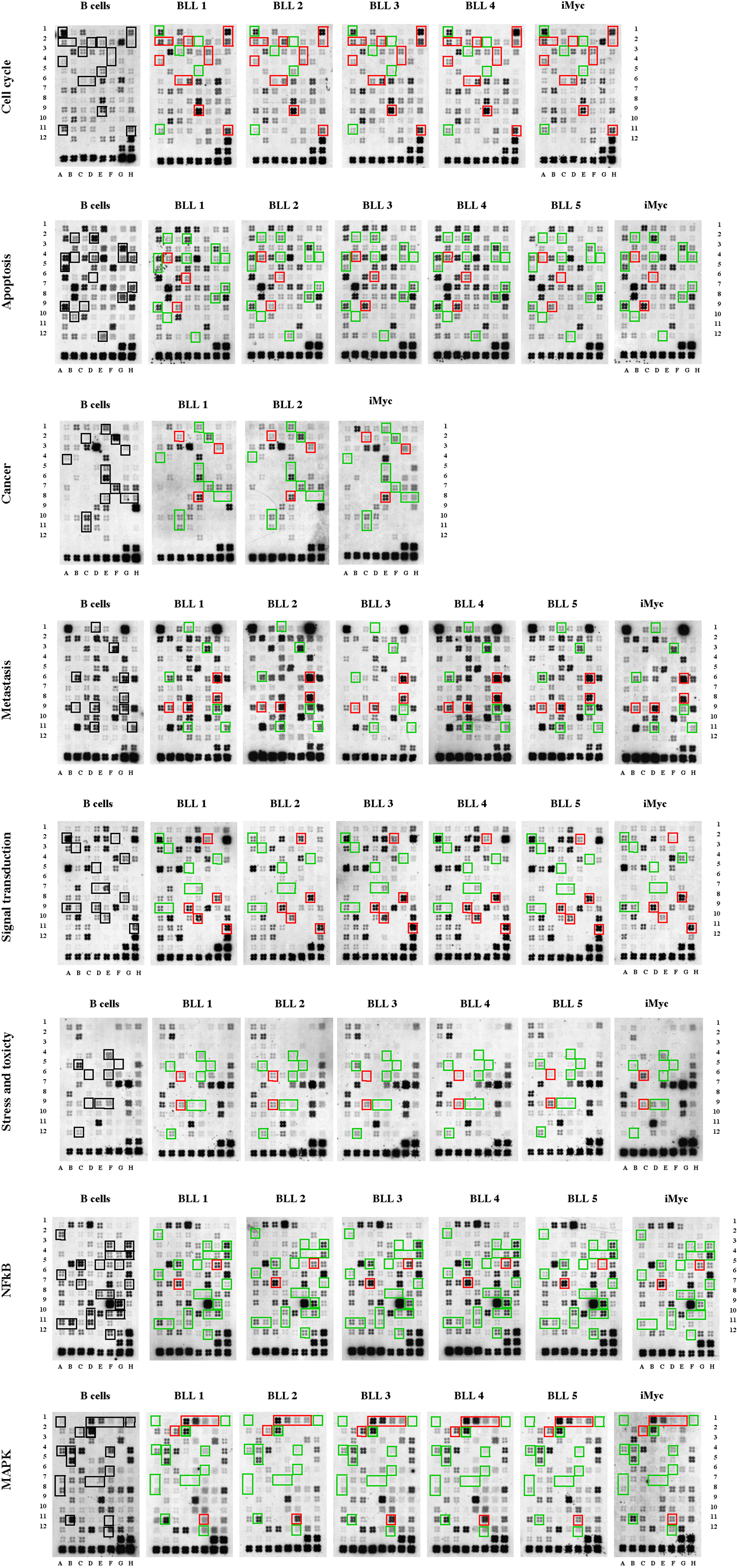

Supplement: Additional File 3 — Images of cDNA gene arrays. [file 1476-4598-4-40-S3.png]

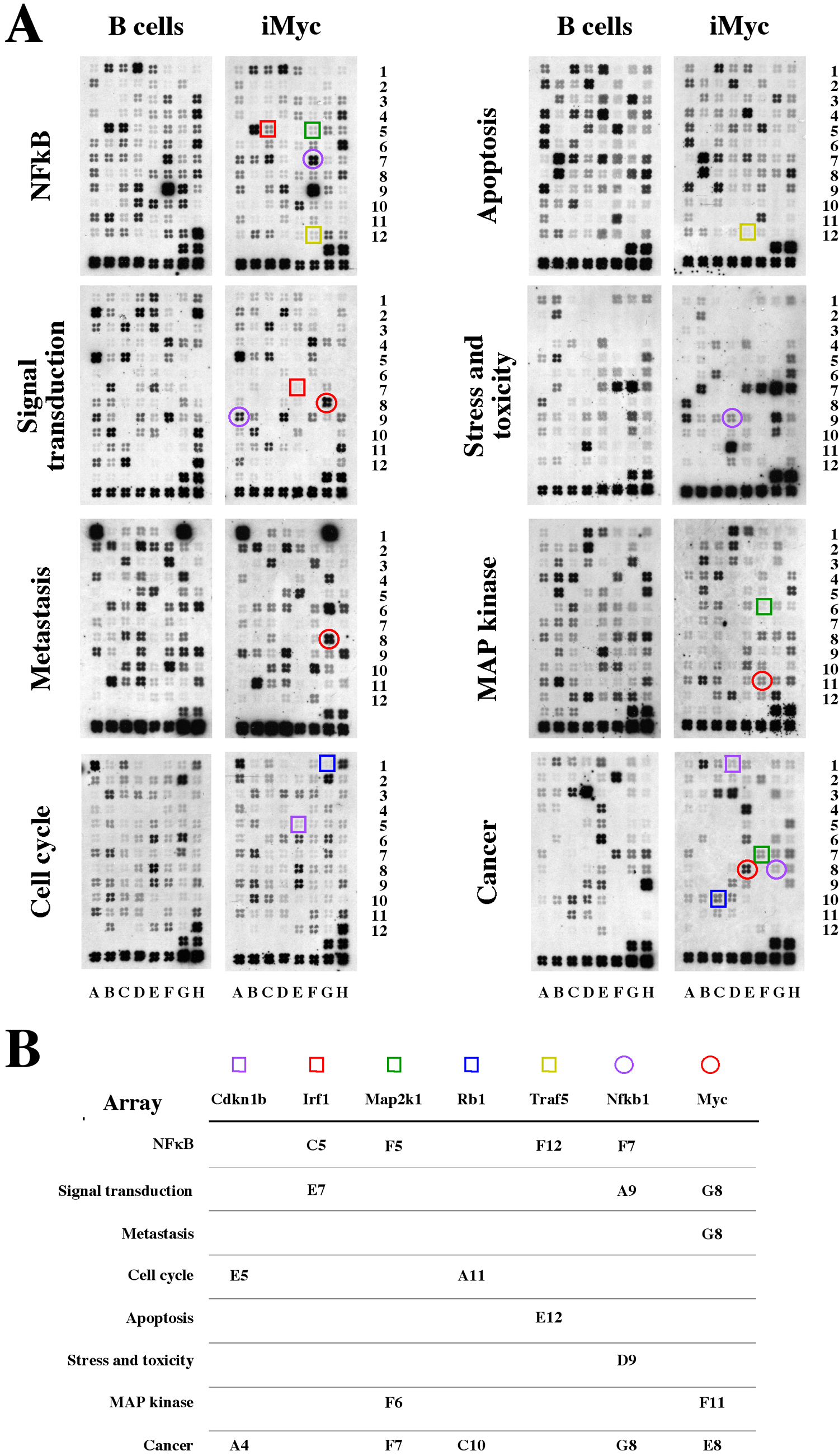

Supplement: Additional File 4 — Images of cDNA gene arrays (panel A) and gene table (panel B). [file 1476-4598-4-40-S4.png]
