# Supplementary material for: Molecular and cytological features of the mouse B-cell lymphoma line iMycEμ-1
Source: Mol Cancer. 2005 Nov 9;4:40. doi: 10.1186/1476-4598-4-40 (PMC1298327; doi:10.1186/1476-4598-4-40)
Supplement: Additional File 5 — Gene list. [file 1476-4598-4-40-S5.doc]

Supplemental Table 1: Differentially regulated genes in iMyc^Eμ^-1 cells and LBL compared to normal B cells

Gene Name Function Grouping

----------------------------------------------------------------------------------------------------------------------------------------------------------------------------------

**Downregulated genes**

*Bcl2a1* B-cell leukemia/lymphoma 2 Apoptosis suppressor Bcl-2 family

related protein A1d

*Birc2* Baculoviral IAP repeat-containing 2 Apoptosis suppressor IAP family

*Cflar* CASP8 and FADD-like apoptosis Apoptosis regulator Death effector domain family

regulator (Cash, Casper, Flip)

*Cdkn1b* Cyclin-dependent kinase inhibitor 1B Cell cycle control G1 phase regulator

(Kip1, p27)

*Grb2* Growth factor receptor bound protein 2 Signal transduction Ras signaling cascade

*Irf1* Interferon regulatory factor 1 Transcription factor NFκB responsive gene

*Jun* Jun oncogene (c-jun) Transcription factor AP-1 component

*Map2k1* Mitogen activated protein kinase kinase 1 Map kinase signaling MKK family

(MAPKK1, MEKK1, Mek1)

*Rb1* Retinoblastoma 1 (Rb) Cell cycle control Rb family

*Ripk1* Receptor (TNFRSF)-interacting serine-threonine Apoptosis regulator Death effector domain family

kinase 1 (RIP)

*Traf5* Tnf receptor-associated factor 5 Apoptosis regulator Traf family

**Upregulated genes**

*Ccna2* Cyclin A2 Cell cycle control S phase regulator

*Ccnb1* Cyclin B1 Cell cycle control S phase regulator

*Myc* Myelocytomatosis oncogene (c-myc) Transcription factor Master regulator

*Nfkb1* Nuclear factor of kappa light chain gene NFκB pathway Rel/NFκB family

enhancer in B-cells 1 (p50/p105)

*Odc* Ornithine decarboxylase Myc target gene Polyamine synthesis
