# Supplementary material for: Molecular and cytological features of the mouse B-cell lymphoma line iMycEμ-1
Source: Mol Cancer. 2005 Nov 9;4:40. doi: 10.1186/1476-4598-4-40 (PMC1298327; doi:10.1186/1476-4598-4-40)
Supplement: Additional File 6 — PCR primers and conditions. [file 1476-4598-4-40-S6.doc]

Supplemental Table 2: RT-PCR primers used for gene array validation.

| Gene | Forward primer sequence | Backward primer sequence |
| --- | --- | --- |
| Odc | CAAAACCAGCAGGCTTCTCTTG | CTCAAACAGCATCCAATCACCC |
| Nfkb1 | ATGATAGCAAAGCCCCGAATGC | TGGAATGTAATCCCACCGTAGG |
| Ccnb1 | CACAGCGGTAGGGATGAAATAGTG | CAATCTCCTTGTCCAGGTAATGCC |
| Ccna2 | AAACAGAGTGTGAAGATGCCCTGG | CAGCCAAGTCAAAAGCAAGGAC |
| Birc2 | ATCAGAGGTCATTGCTGGCGTTC | TTTGCTCGGAAGTTCACAGGGTC |
| Bcl2a1 | ATTTGCCTTTGGGGGTGTTC | TGATAACCATTCTCGTGGGAGCC |
| Cdkn1b | GGGACGGTCGTATCCTTATGAATC | TGGCACACAACCTCTAATCTCAGC |
| Cflar | CACAGAGAATCTACCCACAGAGTGTC | TGAAGCAAGAGGAGGCTCATTG |
| Grb2 | CTTCCCTTCAGGTGTTTTGACG | AACCCACTGCCAAGAGAAATGC |
| Irf1 | GGGACATTGGGATAGGCATACAAC | GCAGCAGTTCTTTGGGAATAGGAC |
| Jun | CCACCGAGACTGTAAAGAAAAGGG | TTGAGGGCATCGTCGTAGAAGGTC |
| Map2k1 | CAATGAGCCTCCTCCAAAACTG | ACAAAGCCAATCCACTTAGGGG |
| Rb1 | TATTTGACACAACCCAGCAGTGCG | TTCGTGGCGTTCTCTCTGTTTC |
| Ripk1 | CAGAGAGGAGGAAAGGAAACGAAG | AGGGTTCAGGTGTTCATCAGTCAG |
| Traf5 | TTCAGCACTGTTCCTTCCAAGC | ACTTCTTCACGGTTTCTGCCAG |
| Gapd | GGTGGAGCCAAACGGGTCATCATCC | CACATTGGGGGTAGGAACACGGAAGG |

## PCR conditions

Denaturation of template DNA: 94°C, 1 min

Annealing of PCR primers: 55°C, 1min

Extension of PCR fragment: 72°C, 1.5 min

30 cycles
